# Sana: democratizing access to quality healthcare using an open mhealth architecture

**Published:** 2012-06-16

**Authors:** Joachim Behar, Alice Newton, George Dafoulas, Leo Anthony Celi, Radhika Chigurupati, Shreesh Naik, Kenneth Paik

**Affiliations:** Engineering Department Oxford, UK; Sana, Computer Science and Artificial Intelligence Laboratory, Massachusetts Institute of Technology, USA; Faculty of Medicine-University of Thessaly, Greece; Sana, Computer Science and Artificial Intelligence Laboratory, Massachusetts Institute of Technology, USA; Sana, Computer Science and Artificial Intelligence Laboratory, Massachusetts Institute of Technology, USA; Sana, Computer Science and Artificial Intelligence Laboratory, Massachusetts Institute of Technology, USA; Sana, Computer Science and Artificial Intelligence Laboratory, Massachusetts Institute of Technology, USA

**Keywords:** mHealth, open source, resource constrained areas

## Abstract

**Introduction:**

Sana is a cell phone-facilitated clinical information system that connects community health workers and medical specialists, to improve screening and diagnostics in resource-constrained settings.

**Aims and objectives:**

The platform allows the transmission of any type of medical data—text, audio, video or photo—from a rural health worker to a remote medical specialist for real-time decision support, and for incorporation into an electronic medical record in order to facilitate care, quality control and allow statistical analysis. By functioning as a portable medical record, Sana also offers the ability to track patients more easily. The point-of-care platform is open source and customizable, allowing doctors to encode new assessments onto smart-phones for a specific application, or to use existing ones. The Sana team partners with universities, social enterprises, governments, NGOs and health organizations in resource-poor countries to assist with implementations of the Sana platform. There are currently eight deployments of Sana in Brazil, Greece, India, the Philippines, Swaziland and Zimbabwe, covering a range of clinical areas.

**Results:**

One of the most urgent problems in resource-poor areas of the world is a shortage of doctors, and especially medical specialists. The biggest implementation of the Sana platform so far has been for the early detection of oral cancer in rural south India. It enables frontline community health workers to screen for precancerous and cancerous lesions by using Sana’s clinical decision support tools in dental hospitals and primary health clinics. Diagnosing oral cancer lesions at an early stage can reduce morbidity, mortality and cost of treatment. Screening processes also increase awareness of risk factors for oral cancer among the population, such as smoking and betel nut chewing. The solution was first implemented in June 2010 and more local health workers and support staff were brought on board in February 2011. By August 2010, up to 6000 people had been screened for oral cancer in the state of Karnataka. 300 patients were identified as high risk and their clinical data, including a photo of the inside of their mouth, were reviewed by an oral cancer specialist at a large tertiary care centre. The plan over the next year is to scale the project to screen one and a half million people in the province of Karnataka.

The developed world, meanwhile, is facing a crisis of an ageing population and a growing burden of chronic disease. In Greece, Sana has been deployed to treat one of the complications of a chronic disease, diabetes. Diabetes continues to be the most common underlying cause of nontraumatic lower extremity amputations, mainly due to foot ulcerations. Sana has developed a mobile health platform for diabetic foot tele-health and a pilot clinical trial to evaluate it is under development in Central Greece. The patients are offered home telehealth consultations during a visit by a specialized nurse, to substitute some of the visits to the outpatient vascular clinic of the hospital.

**Conclusion:**

Oral cancer screening in India enabled the identification of 15 patients with pre-cancerous lesions among the 300 high-risk patients. Evaluation of the cost effectiveness and clinical impact of the technology is underway in deployments such as the project in Greece.

